# Supplementary material for: A Comparative Analysis of Quantitative Metrics of Root Architecture
Source: Plant Phenomics. 2021 Feb 24;2021:6953197. doi: 10.34133/2021/6953197 (PMC8028844; doi:10.34133/2021/6953197)
Supplement: Supplementary 1 — Supplementary Figure S1: representative images of 2D projections of a mazie roots system rotated by 20°, 60°, 100°, 140°, 180°, 220°, 260°, 300°, and 340°. Supplementary Figure S2: cluster heat map of phenotypic traits. Hierarchical clustering of all bean phenotypes was generated using Spearman correlation coefficient of min-max scaled phene values at 40 days (a). The color scale indicates the magnitude of the trait values (blue, low value; red, high value). The numbers indicated on the heat map refer to a representative phenotype in the specific region of the heat map. The corresponding phenotypes are visualized in (b). #: number of axial roots; Axial.Diam: axial root diameter; LRBD: lateral root branching density; Axial.Length: axial root length; Lat.Length: lateral root length; Lat.Diam: lateral root diameter; BW1: basal roots at whorl 1; BW2: basal roots at whorl 2; BW3: basal roots at whorl 3; BW4: basal roots; BW5: basal roots at whorl 5; HBR: hypocotyl-borne roots; PR: primary roots. Supplementary Figure S3: cluster heat map of phenotypic traits. Hierarchical clustering of all maize phenotypes was generated using Spearman correlation coefficient of min-max scaled phene values of at 40 days (a). The color scale indicates the magnitude of the trait values (blue, low value; red, high value). The numbers indicated on the heat map refer to a representative phenotype in the specific region of the heat map. The corresponding phenotypes are visualized in (b). #: number of axial roots; Axial.Diam: axial root diameter; LRBD: lateral root branching density; Axial.Length: axial root length; Lat.Length: lateral root length; Lat.Diam: lateral root diameter; NR1: nodal roots at position 1; NR2: nodal roots at position 2; NR3: nodal roots at position 3; NR4: nodal roots at position 4; SR: seminal roots; PR: primary roots. Supplementary Figure S4: trait dynamics of bean root phenotypes over 30 days of growth from day 10 to 40. Change in estimates of phenes (a). Change in esti [file 6953197.f1.pdf]

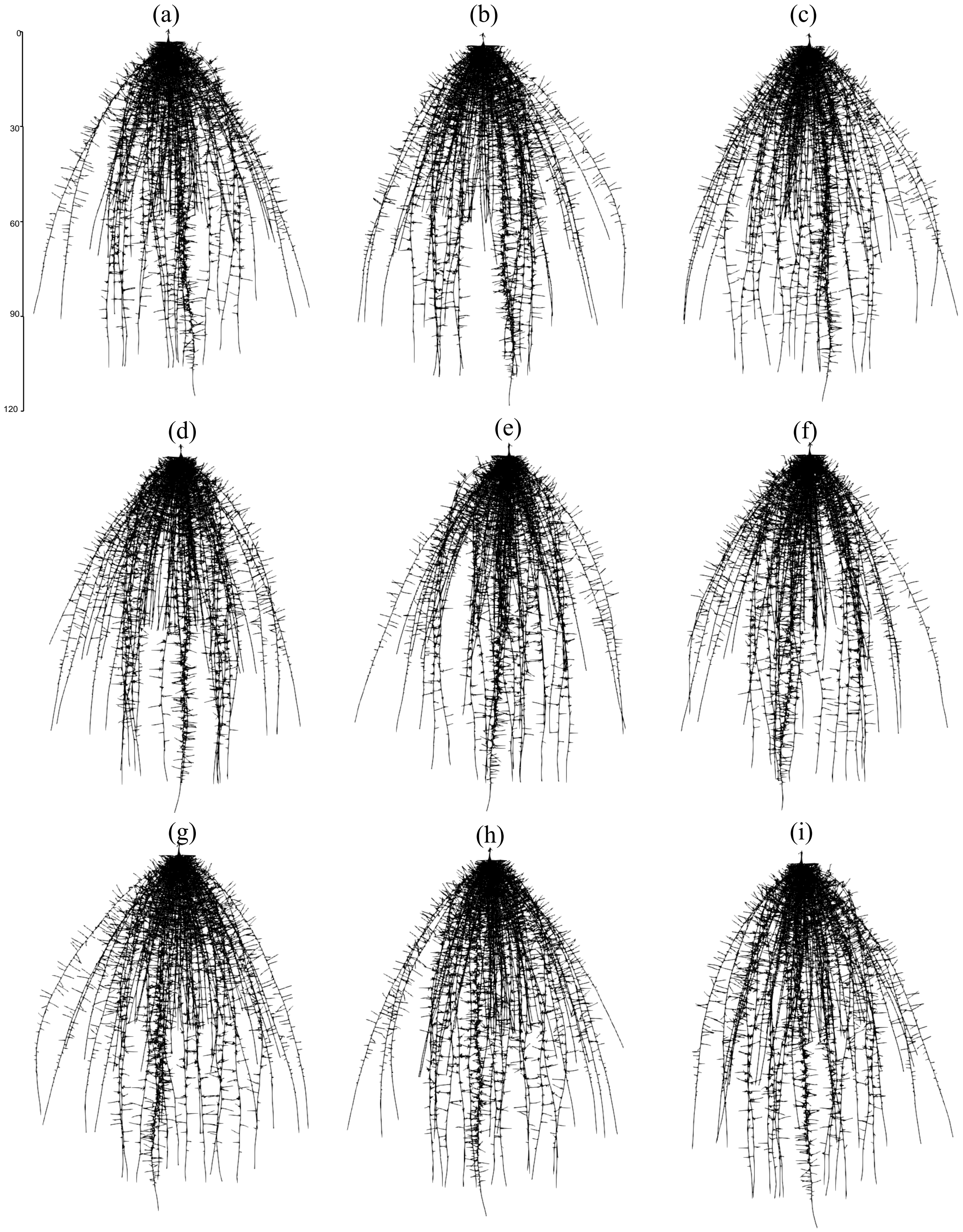

Supplementary Figure 1(a)-1(i): Representative images of 2D projections of a maize root system rotated by 20°, 60°, 100°, 140°, 180°, 220°, 260°, 300°, 340°.

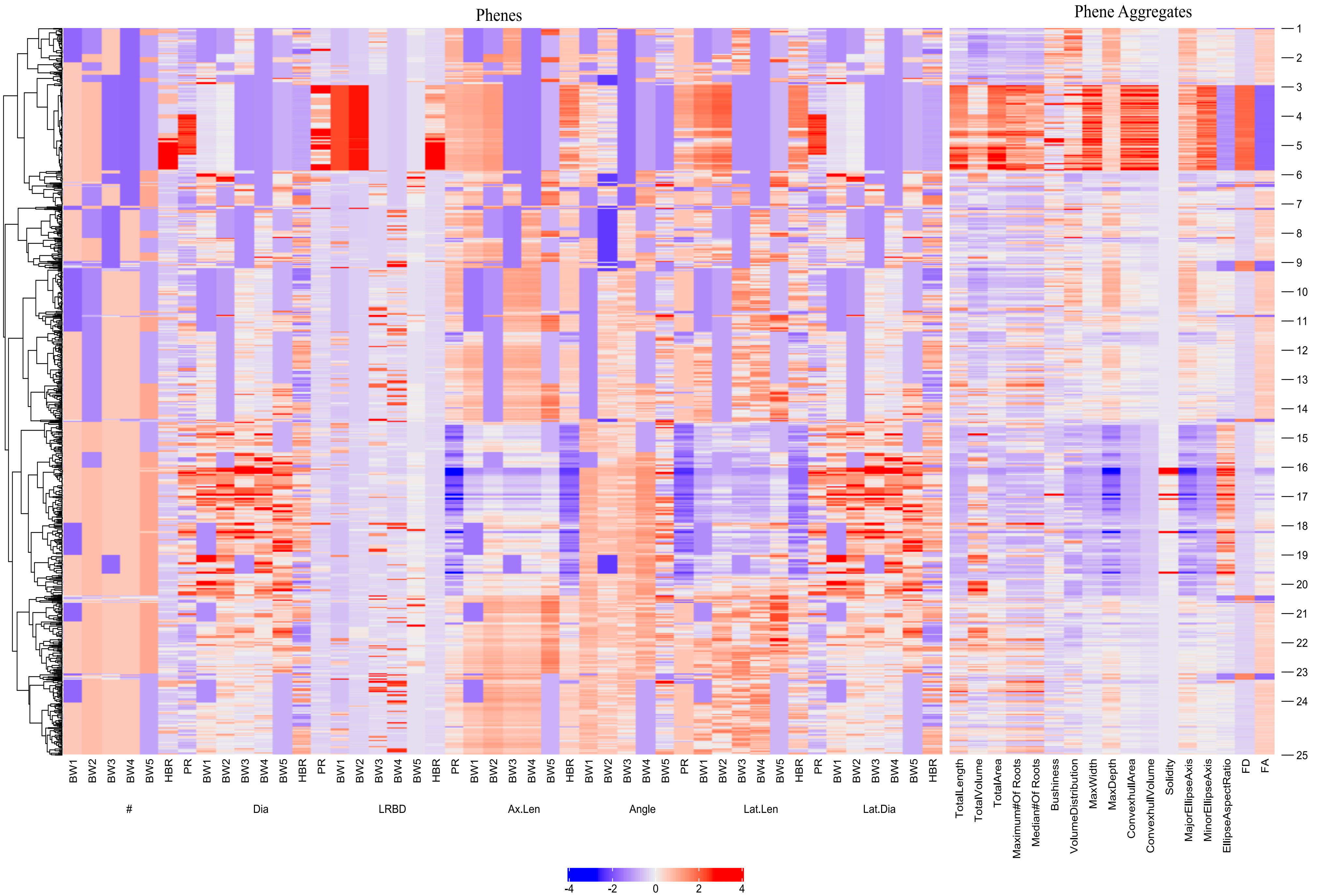
Phenes
Phene Aggregates
BW1 BW2 BW3 BW4 BW5 HBR PR BW1 BW2 BW3 BW4 BW5 HBR PR BW1 BW2 BW3 BW4 BW5 HBR PR BW1 BW2 BW3 BW4 BW5 HBR BW1 BW2 BW3 BW4 BW5 PR BW1 BW2 BW3 BW4 BW5 HBR PR BW1 BW2 BW3 BW4 BW5 HBR
#
Dia
LRBD
Ax.Len
Angle
Lat.Len
Lat.Dia
TotalLength
TotalVolume
TotalArea
Maximum#Of Roots
Median#Of Roots
Bushiness
VolumeDistribution
MaxWidth
MaxDepth
ConvexhullArea
ConvexhullVolume
Solidity
MajorEllipseAxis
MinorEllipseAxis
EllipseAspectRatio
FD
FA
1
2
3
4
5
6
7
8
9
10
11
12
13
14
15
16
17
18
19
20
21
22
23
24
25
-4
-2
0
2
4
(a)

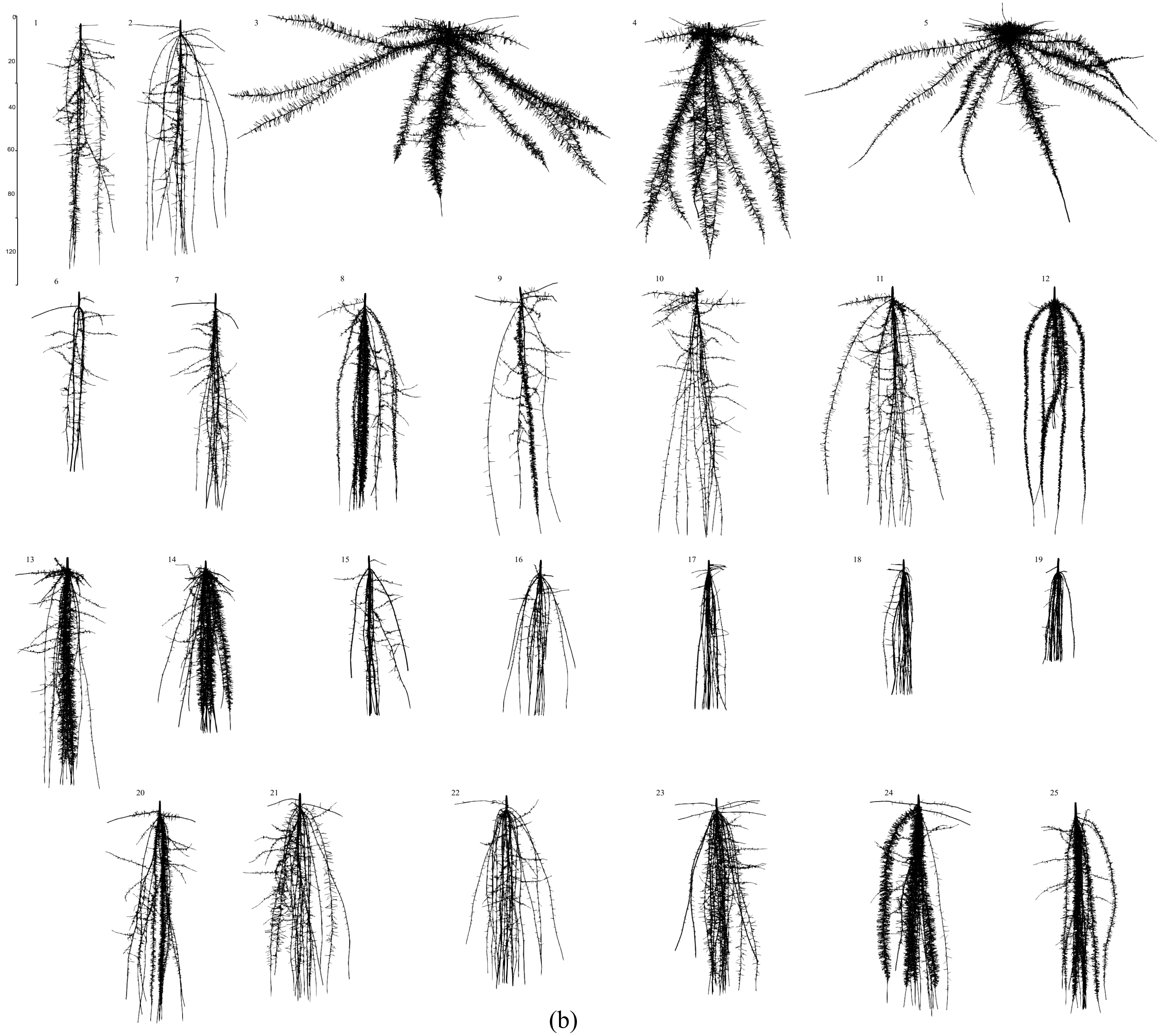

(b)

Supplementary Figure 2: Cluster heatmap of phenotypic traits. Hierarchical clustering of all bean phenotypes was generated using Spearman correlation coefficient of max-min scaled phene values at 40 days (a). The color scale indicates the magnitude of the trait values (blue, low value; red, high value).
The numbers indicated on the heatmap refer to a representative phenotype in the specific region of the heatmap. The corresponding phenotypes are visualized in (b).
# - Number of roots; Axial.Diam - axial root diameter; LRBD - lateral root branching density; Axial.Length - axial root length; Lat.Length- lateral root length; Lat.Diam - lateral root diameter; BW1 - basal roots at whorl 1; BW2 - basal roots at whorl 2; BW3 - basal roots at whorl 3; BW4 - basal roots at whorl 4; BW5 - basal roots at whorl 5; HBR - hypocotyl-borne roots; PR - primary root.

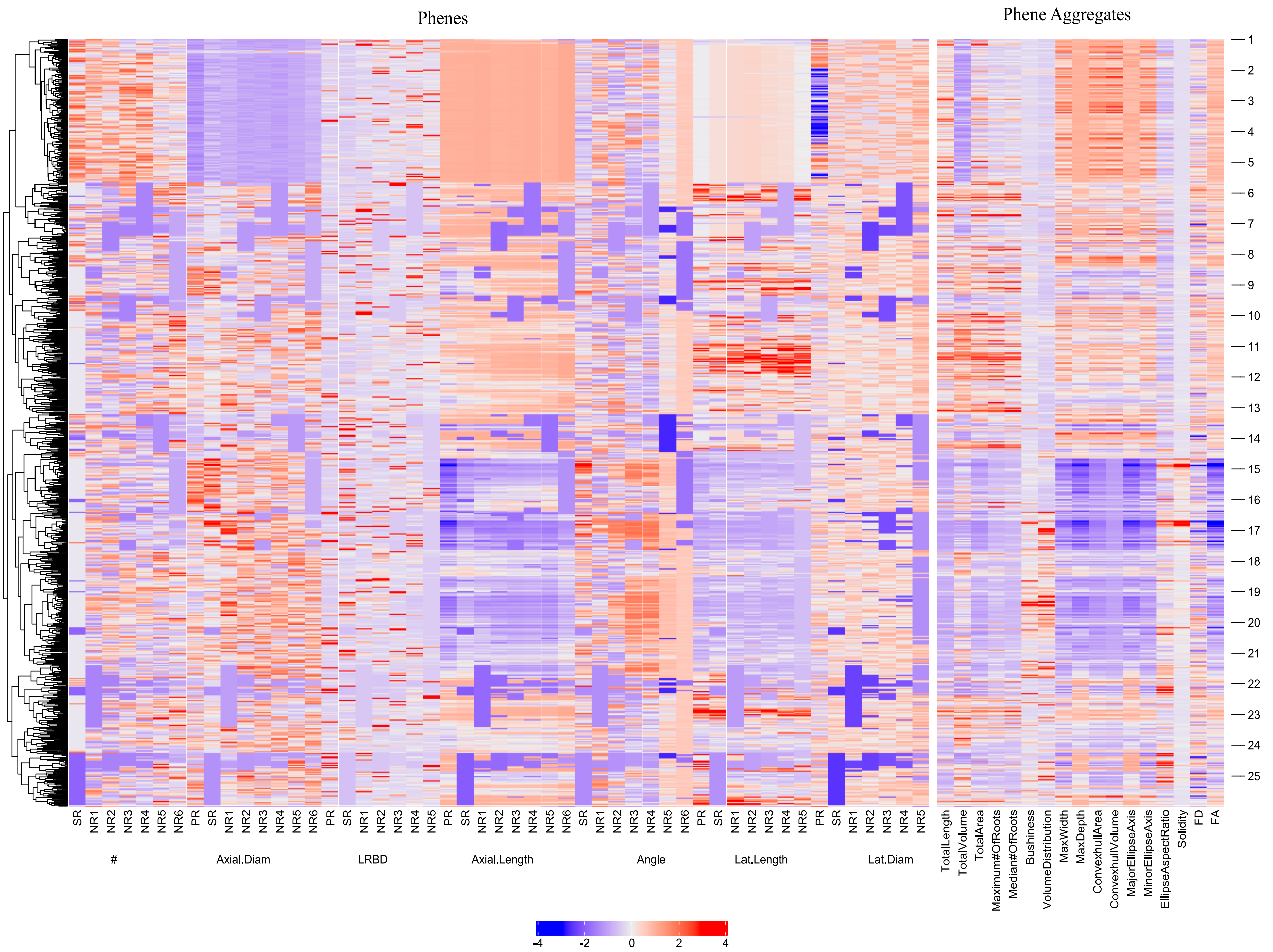
Phenes
Phene Aggregates
SR NR1 NR2 NR3 NR4 NR5 NR6
PR SR NR1 NR2 NR3 NR4 NR5 NR6
PR SR NR1 NR2 NR3 NR4 NR5
PR SR NR1 NR2 NR3 NR4 NR5 NR6
SR NR1 NR2 NR3 NR4 NR5 NR6
PR SR NR1 NR2 NR3 NR4 NR5
PR SR NR1 NR2 NR3 NR4 NR5
#
Axial.Diam
LRBD
Axial.Length
Angle
Lat.Length
Lat.Diam
TotalLength
TotalVolume
TotalArea
Maximum#OfRoots
Median#OfRoots
Bushiness
VolumeDistribution
MaxWidth
MaxDepth
ConvexhullArea
ConvexhullVolume
MajorEllipseAxis
MinorEllipseAxis
EllipseAspectRatio
Solidity
FD
FA
1 2 3 4 5 6 7 8 9 10 11 12 13 14 15 16 17 18 19 20 21 22 23 24 25
-4 -2 0 2 4

(a)

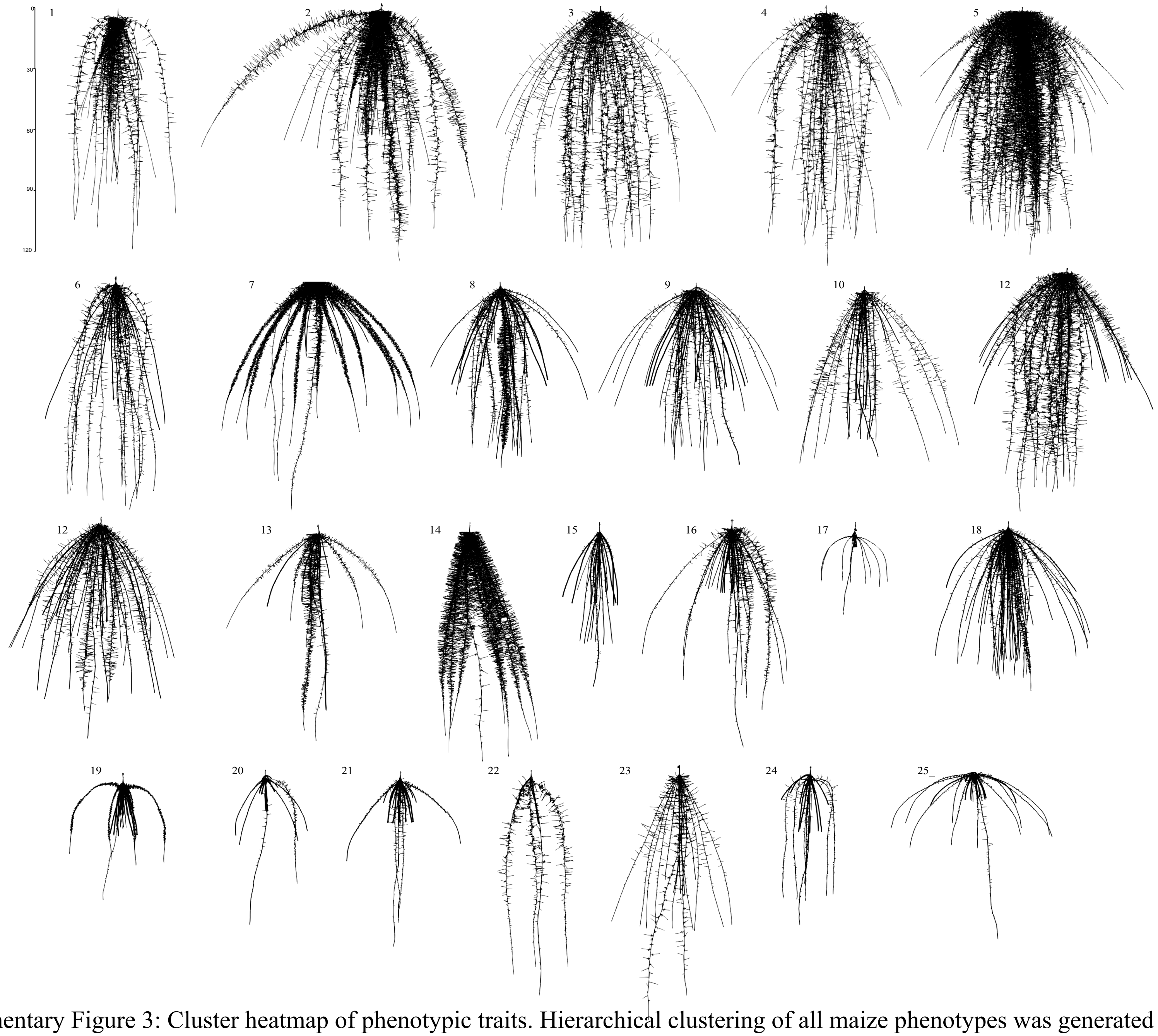

Supplementary Figure 3: Cluster heatmap of phenotypic traits. Hierarchical clustering of all maize phenotypes was generated using Spearman correlation coefficient of max-min scaled phene values at 40 days (a). The color scale indicates the magnitude of the trait values (blue, low value; red, high value).
The numbers indicated on the heatmap refer to a representative phenotype in the specific region of the heatmap. The corresponding phenotypes are visualized in (b).
# - Number of roots; Axial.Diam - axial root diameter; LRBD - lateral root branching density; Axial.Length - axial root length; Lat.Length- lateral root length; Lat.Diam - lateral root diameter; NR1 - nodal roots at position 1; NR2 - nodal roots at position 2; NR3 - nodal roots at position 3; NR4 - nodal roots at position 4 ; SR - seminal roots; PR - primary root.

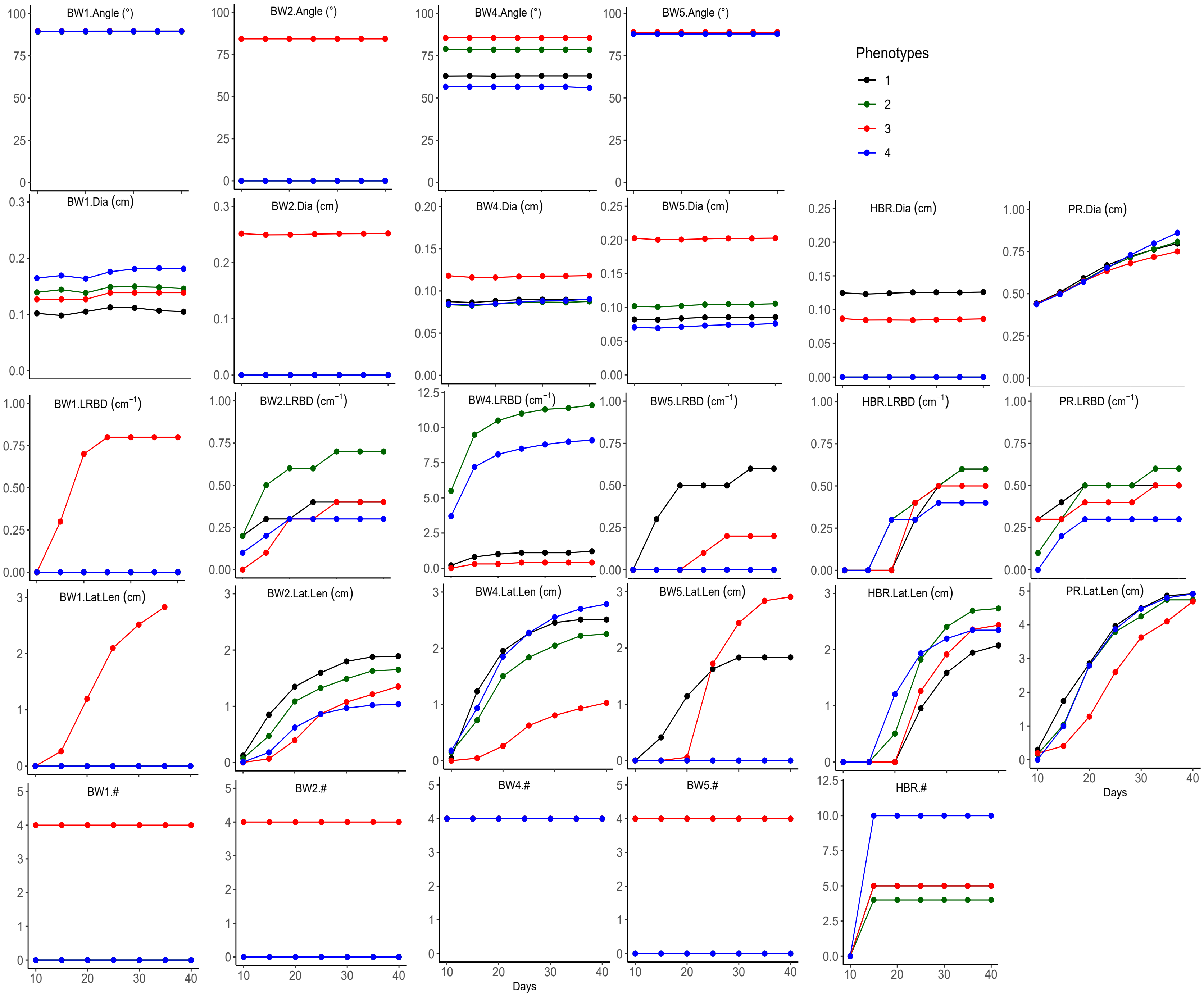
BW1.Angle (°)
BW2.Angle (°)
BW4.Angle (°)
BW5.Angle (°)
Phenotypes
1
2
3
4
BW1.Dia (cm)
BW2.Dia (cm)
BW4.Dia (cm)
BW5.Dia (cm)
HBR.Dia (cm)
PR.Dia (cm)
BW1.LRBD (cm^{-1})
BW2.LRBD (cm^{-1})
BW4.LRBD (cm^{-1})
BW5.LRBD (cm^{-1})
HBR.LRBD (cm^{-1})
PR.LRBD (cm^{-1})
BW1.Lat.Len (cm)
BW2.Lat.Len (cm)
BW4.Lat.Len (cm)
BW5.Lat.Len (cm)
HBR.Lat.Len (cm)
PR.Lat.Len (cm)
BW1.#
BW2.#
BW4.#
BW5.#
HBR.#
Days

(a)

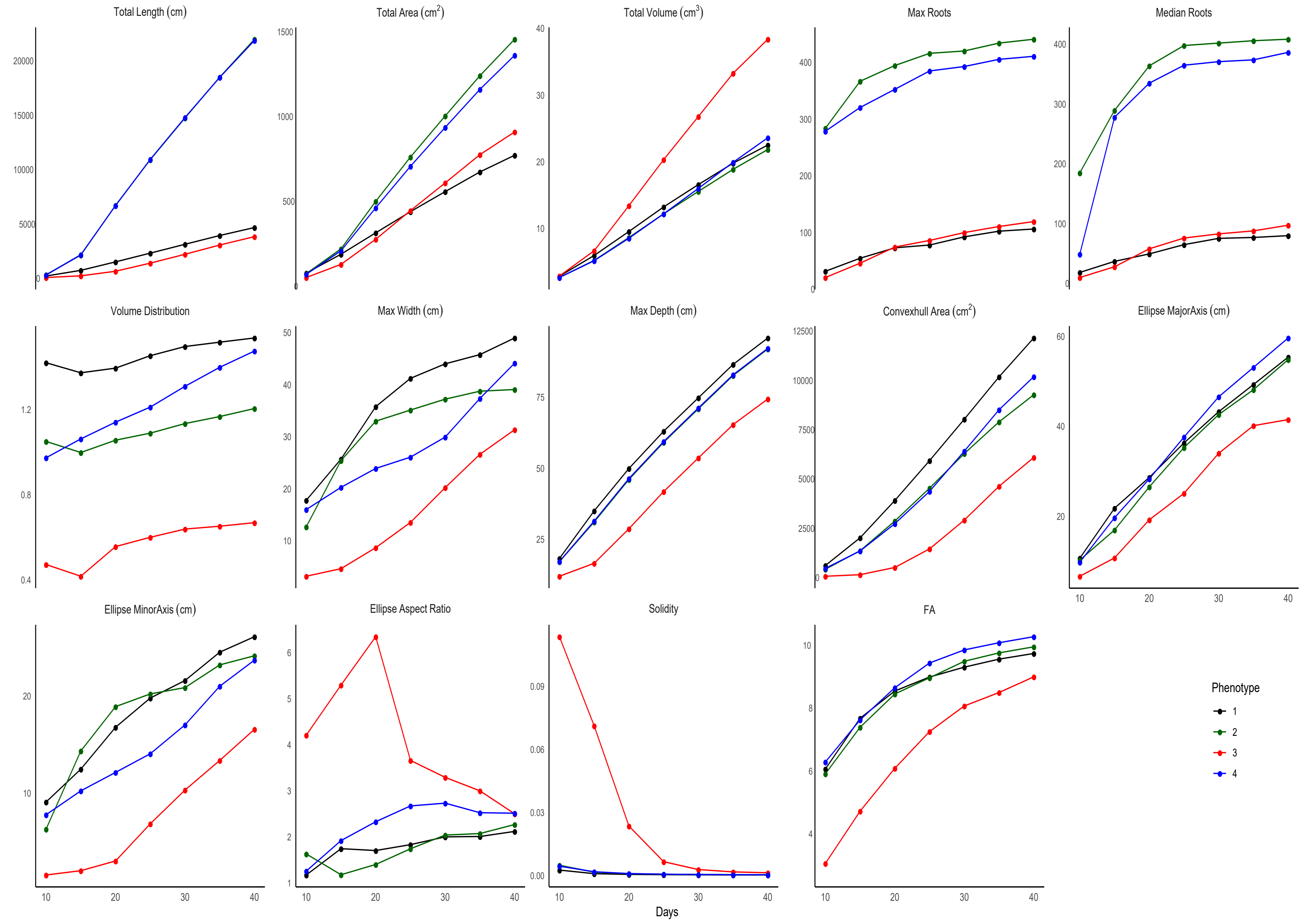

(b)

Supplementary Figure 4: Trait dynamics of bean root phenotypes over 30 days of growth from day 10 to day 40. Change in estimates of phenes (a). Change in estimates of the phene aggregates (b). BW1 - basal roots at whorl 1; BW2 - basal roots at whorl 2; BW4 - basal roots at whorl 4; BW5 - basal roots at whorl 5; HBR - hypocotyl-borne roots; PR - primary root; Dia - axial root diameter; LRBD - lateral root branching density; Lat.Len - lateral root length; # - number of axial roots; FA - fractal abundance.

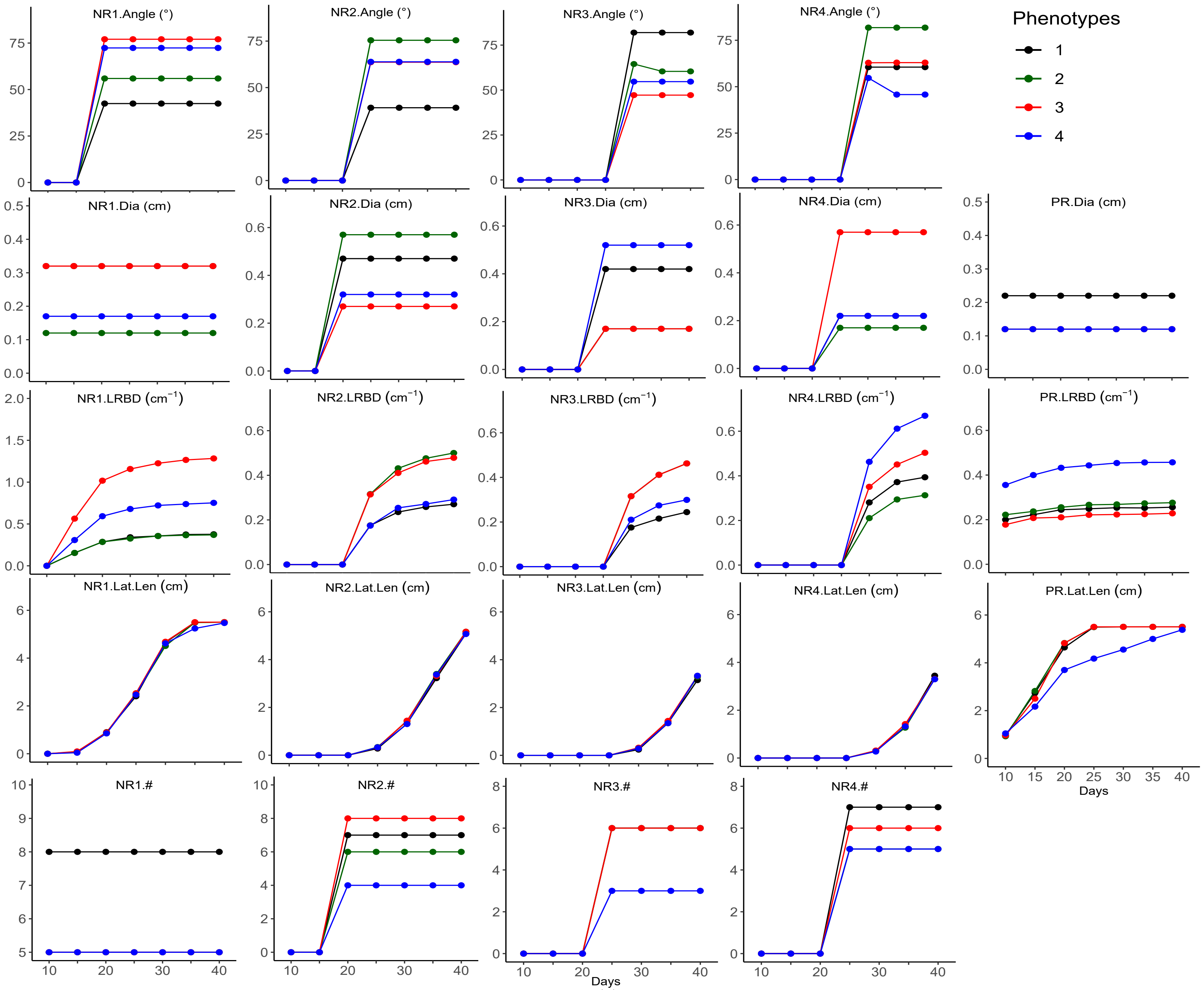

Phenotypes
1
2
3
4
NR1.Angle (°)
NR2.Angle (°)
NR3.Angle (°)
NR4.Angle (°)
NR1.Dia (cm)
NR2.Dia (cm)
NR3.Dia (cm)
NR4.Dia (cm)
PR.Dia (cm)
NR1.LRBD (cm^{-1})
NR2.LRBD (cm^{-1})
NR3.LRBD (cm^{-1})
NR4.LRBD (cm^{-1})
PR.LRBD (cm^{-1})
NR1.Lat.Len (cm)
NR2.Lat.Len (cm)
NR3.Lat.Len (cm)
NR4.Lat.Len (cm)
PR.Lat.Len (cm)
NR1.#
NR2.#
NR3.#
NR4.#
Days
Days

(a)

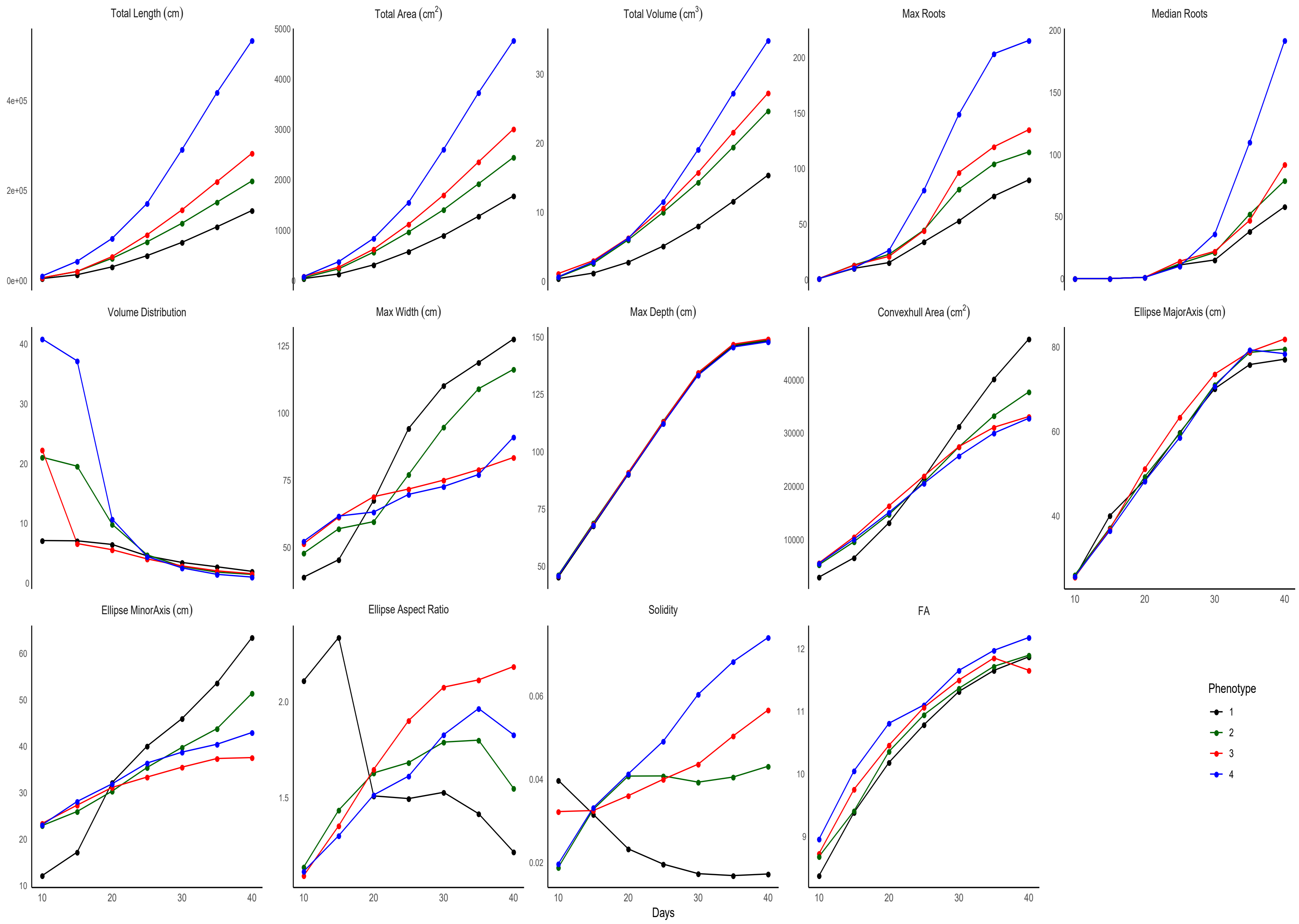

Supplementary Figure 5: Trait dynamics of maize root phenotypes over 30 days of growth from day 10 to day 40. Change in estimates of phenes (a). Change in estimates of the phene aggregates (b). NR1 - nodal roots at position 1; NR2 - nodal roots at position 2; NR3 - nodal roots at position 3; NR4 - nodal roots at position 4; PR - primary root; Dia - axial root diameter; LRBD - lateral root branching density; Lat.Len - lateral root length; # - number of axial roots; FA - fractal abundance.
