# Supplementary material for: A Comparative Analysis of Quantitative Metrics of Root Architecture
Source: Plant Phenomics. 2021 Feb 24;2021:6953197. doi: 10.34133/2021/6953197 (PMC8028844; doi:10.34133/2021/6953197)
Supplement: Supplementary 2 — Supplementary Table S1: range of input values for generating bean root phenotypes. PR: primary root; HBR: hypocotyl-borne-root; BW: basal whorl; BW1, BW2, BW3, BW4, and BW5 refer to the position of the basal whorl counted from basipetal to acropetal position; Dia: axial root diameter; Lat.Dia: lateral root diameter; LRBD: lateral root branching density. Supplementary Table S2: range of input values for generating maize root phenotypes. PR: primary root; SR: seminal root; NR: nodal root; NR1, NR2, NR3, and NR4 refer to the nodal root position; Dia: axial root diameter; Lat.Dia: lateral root diameter; LRBD: lateral root branching density. ∗NR at different positions was considered to have similar parameters. [file 6953197.f2.pdf]

Supplementary Material 2

Supplementary Table S1: Range of input values for generating bean root phenotypes. PR – primary root; HBR- Hypocotyl-Borne-Root; BW – Basal Whorl; BW1, BW2, BW3, BW4, BW5 refer to the position of the basal whorl counted from basipetal to acropetal position; Dia – axial root diameter; Lat.Dia – lateral root diameter; LRBD – lateral root branching density.

	Units	Min	Max	References
Number.BW1	NA	0	4	Miguel et al., 2013
Number.BW2	NA	0	4	
Number.BW3	NA	0	4	
Number.BW4	NA	0	4	
Number.BW5	NA	0	4	
Number.HBR	NA	0	30	Miller et al., 2003
PR.Dia	cm	0.08	0.45	Henry et al., 2009
BW1.Dia	cm	0	0.45	
BW2.Dia	cm	0	0.45	
BW3.Dia	cm	0	0.45	
BW4.Dia	cm	0	0.45	
BW5.Dia	cm	0	0.45	
HBR.Dia	cm	0	0.45	
BW1.Lat.Dia	cm	0	0.03	
BW2.Lat.Dia	cm	0	0.03	
BW3.Lat.Dia	cm	0	0.03	
BW4.Lat.Dia	cm	0	0.03	
BW5.Lat.Dia	cm	0	0.03	
HBR.Lat.Dia	cm	0	0.03	
PR.Lat.Dia	cm	0	0.03	
BW1.LRBD	cm^{-1}	0	40	Miller et al., 2003
BW2.LRBD	cm^{-1}	0	40	
BW3.LRBD	cm^{-1}	0	40	
BW4.LRBD	cm^{-1}	0	40	
BW5.LRBD	cm^{-1}	0	40	
PR.LRBD	cm^{-1}	0	40	
HBR.LRBD	cm^{-1}	0	40	
BW1.Angle	degree	0	90	Miguel et al., 2013
BW2.Angle	degree	0	90	
BW3.Angle	degree	0	90	
BW4.Angle	degree	0	90	
BW5.Angle	degree	0	90	

Supplementary Table S2: Range of input values for generating maize root phenotypes. PR - Primary Root; SR -Seminal Root; NR-Nodal Root; NR1, NR2, NR3, NR4 refer to the nodal root position; Dia – axial root diameter; Lat.Dia – lateral root diameter; LRBD – lateral root branching density. *NR at different positions were considered to have similar parameters.

	Units	Min	Max	References
Number.SR	NA	0	12	Hochholdinger and Tuberosa, 2009
Number.NR1	NA	0	12	Burton et al., 2013; York and Lynch, 2015
Number.NR2	NA	0	12	
Number.NR3	NA	0	12	
Number.NR4	NA	0	12	
PR.Dia	cm	0.08	0.6	Burton et al., 2013; Burton et al., 2014; York and Lynch, 2015
SR.Dia	cm	0	0.6	
NR1.Dia	cm	0	0.6	
NR2.Dia	cm	0	0.6	
NR3.Dia	cm	0	0.6	
NR4.Dia	cm	0	0.6	
PR.Lat.Dia	cm	0	0.05	
SR.Lat.Dia	cm	0	0.05	
NR1.Lat.Dia	cm	0	0.05	
NR2.Lat.Dia	cm	0	0.05	
NR3.Lat.Dia	cm	0	0.05	
NR4.Lat.Dia	cm	0	0.05	
PR.LRBD	cm^{-1}	0	40	Postma et al., 2014; York and Lynch, 2015
SR.LRBD	cm^{-1}	0	40	
NR1.LRBD	cm^{-1}	0	40	
NR2.LRBD	cm^{-1}	0	40	
NR3.LRBD	cm^{-1}	0	40	
NR4.LRBD	cm^{-1}	0	40	
SR.Angle	degree	0	90	Liao et al., 2004; Zhu et al., 2005
NR1.Angle	degree	0	90	
NR2.Angle	degree	0	90	
NR3.Angle	degree	0	90	
NR4.Angle	degree	0	90	

Citations

Burton AL, Brown KM, Lynch JP (2013) Phenotypic Diversity of Root Anatomical and Architectural Traits in Species. Crop Sci **53**: 1042-1055

Burton AL, Johnson JM, Foerster JM, Hirsch CN, Buell CR, Hanlon MT, Kaeppler SM, Brown KM, Lynch JP, Hanlon MT, et al (2014) QTL mapping and phenotypic variation for root architectural traits in maize (Zea mays L.). Theor Appl Genet **127**: 2293-2311

Henry A, Kleinman PJA, Lynch JP (2009) Phosphorus runoff from a phosphorus deficient soil under common bean (Phaseolus vulgaris L.) and soybean (Glycine max L.) genotypes with contrasting root architecture. Plant Soil **317**: 1–16

Hochholdinger F, Tuberosa R (2009) Genetic and genomic dissection of maize root development and architecture. Curr Opin Plant Biol **12**: 172–177

Liao H, Yan X, Rubio G, Beebe SE, Blair MW, Lynch JP (2004) Genetic mapping of basal root gravitropism and phosphorus acquisition efficiency in common bean. Funct Plant Biol **31**: 959–970

Miguel MA, Widrig A, Vieira RF, Brown KM, Lynch JP (2013) Basal root whorl number: A modulator of phosphorus acquisition in common bean (Phaseolus vulgaris). Ann Bot **112**: 973–982

Miller CR, Ochoa I, Nielsen KL, Beck D, Lynch JP (2003) Genetic variation for adventitious rooting in response to low phosphorus availability: Potential utility for phosphorus acquisition from stratified soils. Funct Plant Biol **30**: 973–985

Postma JA, Dathe A, Lynch JP (2014) The optimal lateral root branching density for maize depends on nitrogen and phosphorus availability. Plant Physiol **166**: 590-602

York LM, Lynch JP (2015) Intensive field phenotyping of maize (Zea mays L.) root crowns identifies phenes and phene integration associated with plant growth and nitrogen acquisition. J Exp Bot **66**: 5493–5505

Zhu J, Kaeppler SM, Lynch JP (2005) Topsoil foraging and phosphorus acquisition efficiency in maize (Zea mays). Funct Plant Biol **32**: 749
